# Splenectomy and emerging novel treatments in rare inherited hemolytic anemias

**DOI:** 10.1097/HS9.0000000000000190

**Published:** 2019-06-30

**Authors:** Joanne Yacobovich, Hannah Tamary

**Affiliations:** Hematology Oncology Division, Hematology Unit, Schneider Children's Medical Center of Israel, Petah Tikva, Israel

**Take home messages**

- Splenectomy should be considered in severely affected patients with inherited hemolytic anemia.
- Splenectomy is associated with infectious and thrombotic complications.
- Separately for each disorder, prior to the decision to proceed to splenectomy, the efficacy of this procedure in reliving the anemia, and the already described thrombotic complications should be considered.

## Introduction

As abnormal or damaged red blood cells passing through the spleen red pulp are efficiently removed by the splenic macrophage system, splenectomy has been suggested as a possible therapeutic approach to manage severely affected patients with inherited hemolytic anemias. The efficacy of splenectomy in many of these disorders has yet to be determined. Additionally, concern remains regarding short- and long-term infectious and thrombotic complications.^1▪^ In view of the variable efficacy and possible complications of this procedure, expert recommendations for splenectomy in hereditary hemolytic anemias have been recently published by the EHA Working Study group on Red cells and Iron (EHA-WG-RI).^2▪^ In this short manuscript, we will review the complications of splenectomy in 2 inherited hemolytic anemias (pyruvate kinase deficiency [PKD] and hereditary stomatocytosis [HSt]), as well as emerging alternative future therapeutic options in those disorders. Splenectomy in hereditary spherocytosis (HS) is discussed in the previous chapter and only summary of the indications for this procedure is summarized in Table ^1^.

**Table 1:**
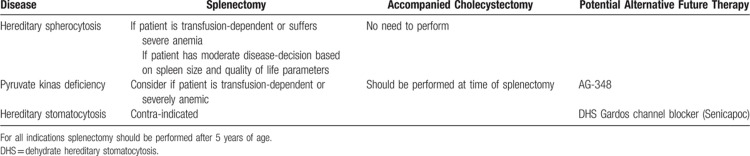
Inherited Hemolytic Disorders: Splenectomy, Cholecystectomy, and Future Alternative Therapy

## Current state of the art

### Splenectomy complications

#### Postsplenectomy infections

Due to the role of the spleen in immune competence and blood filtration, following splenectomy there is a risk of overwhelming infection (OPSI) which is highest with encapsulated organisms such as *Streptococcus pneumoniae*, *Neisseria meningitidis*, and *Haemophilus influenzae*.^3^ Asplenia is also an important risk factor for serious infections with *Plasmodium*, *Capnocytophaga canimorsus*, and *cynodegmi* (after an animal bite), Babesia (after a tick bite), and *Bordetella holmesii.*^4^ An elevated risk of OPSI probably remains for life.^5^ Due to the high risk of this complication at a young age, splenectomy should not normally be performed before 5 years of age. Recent studies suggested that OPSI occurs in about 4% to 7% of patients with hematological disorders while most of them were found to be nonimmunized.^6^ The addition of conjugated pneumococcal and meningococcal vaccines, as well as meningococcal B recombinant vaccine, accompanied by efforts to increase adherence to vaccination protocols, may further reduce the risk of OPSI. Guidelines regarding prevention and treatment of infections in splenectomized patients have been recently published by the American Academy of Pediatrics (Red Book 31st edition, 2018); the reader is referred to this publication for detailed instructions.

### Postsplenectomy thromboembolic complications

Thromboembolic events in hemolytic anemias following splenectomy have been sporadically reported. Those reports describe acute splenic and portal vein thrombosis (SPVT), and also, delayed life-long events.

#### Acute splenic and portal vein thrombosis

This is an early life-threatening complication, which can lead to bowel ischemia and/or portal hypertension. This complication is probably due to stasis in the splenic vein remnant.^7^ Screening with contrast-enhanced computed tomography showed a median time of 6 days between splenectomy and the appearance of asymptomatic SPVT.^8^ The EHA-WG-RI recommended that Doppler ultrasound screening for SPVT should be carried out on day 7 postsplenectomy.^2▪^

#### Venous thromboembolism and arterial pulmonary hypertension

Deep vein thrombosis, pulmonary emboli, and Pulmonary Arterial Hypertension have sporadically been described following splenectomy in patients with HS, PKD, HSt, and unstable hemoglobin.^9^–^13^ The etiology of these complications is probably multifactorial and includes increased platelet number and activation, leukocytosis, activation of the endothelium, altered lipid profile, and NO consumption due to continued intravascular hemolysis.^1▪^ More studies are required to better define the risk of thromboembolism related to splenectomy.

## Splenectomy in pyruvate kinase deficiency

PKD is the most common glycolytic defect leading to lack of energy to support membrane RBC structure and ion transport. Splenectomy only partially ameliorates the anemia but is usually beneficial in decreasing the transfusion need. The recently published Pyruvate Kinase Deficiency Natural History Study that enrolled 278 PKD patients suggested that 59% of patients underwent splenectomy.^14▪^ Eleven percent of those developed thrombosis following splenectomy compared to no occurrences of thrombosis in patients who were not splenectomized. Due to postsplenectomy residual hemolysis, 48% of patients who had a splenectomy without simultaneous cholecystectomy later required a cholecystectomy. EHA-WG-RI therefore suggested that splenectomy should be considered in patients with PKD who are transfusion-dependent or intolerant of the anemia; and that cholecystectomy should always accompany splenectomy.^2▪^

## Splenectomy in hereditary stomatocytosis

HSt is a dominant disorder including both dehydrated (DHS) and overhydrated (OHS) types, with alteration of the RBC membrane permeability to monovalent cations (Na^+^ and K^+^) and, with a consequent alteration in the intracellular cationic content and in red cell volume. Recent studies suggested that DHS is mainly caused by gain of function mutations in the *PIEZO1* gene encoding for a cationic channel. *PIEZO1* mutations result in significant uptake of Na^+^, K^+^ loss, and Ca^++^ influx leading to activation of the Gardos channel and water loss. Few cases of DHS were recently found to be caused by activating mutations in *KCNN4* gene encoding for the Gardos channel itself.^15▪^

Splenectomy is ineffective in DHS and only partially effective in OHS. In addition, this procedure was found to be associated with severely increased risk of thromboembolic complications.^12^ Therefore, it has been suggested by the EHA-WG-RI that splenectomy in patients with HSt is probably contra-indicated.^2▪^

## Future prospectives

New therapies are emerging as alternative to splenectomy in PKD and DHS. An oral pharmacologic activator of RBC pyruvate kinase, AG-348, is currently in clinical trials.^16▪^ Early results from a phase II trial in patients demonstrated increased hemoglobin in a significant subset of patients with hemolysis.^17^ Patients with at least 1 missense mutation were found to be more likely to respond. Preclinical studies also suggest that PKD may be a candidate disease for gene therapy.^18^

Gardos channel blockers such as Senicapoc have been shown to prevent in vitro DHS RBC dehydration due to *PIEZO1* or *KCNN4* activating mutations.^19^ A phase III clinical trial evaluating the efficiency of Senicapoc to reduce the frequency of sickle cell pain crises showed that although Senicapoc administration did improve erythrocyte rehydration, there was no efficacy in reducing vaso-occlusive crises.^20^ Nevertheless, Senicapoc administration was well-tolerated and showed no significant toxicity. These results point to a possible therapeutic effect of Senicapoc in DHS and future studies are awaited.
